# Association among Adherence to the Mediterranean Diet, Cardiorespiratory Fitness, Cardiovascular, Obesity, and Anthropometric Variables of Overweight and Obese Middle-Aged and Older Adults

**DOI:** 10.3390/nu12092750

**Published:** 2020-09-10

**Authors:** Pablo J. Marcos-Pardo, Noelia González-Gálvez, Alejandro Espeso-García, Tomás Abelleira-Lamela, Abraham López-Vivancos, Raquel Vaquero-Cristóbal

**Affiliations:** 1 Research Group on Health, Physical Activity, Fitness and Motor Behaviour (GISAFFCOM), Catholic University San Antonio of Murcia, Campus de los Jerónimos, Guadalupe, 30107 Murcia, Spain; pmarcos@ucam.edu (P.J.M.-P.); aespeso@ucam.edu (A.E.-G.); tabelleira@alu.ucam.edu (T.A.-L.); alvivancos@ucam.edu (A.L.-V.); 2 Active Aging, Exercise and Health/HEALTHY-AGE Network, Consejo Superior de Deportes (CSD), Ministry of Culture and Sport of Spain, 28040 Madrid, Spain; rvaquero@ucam.edu; 3 Sports Injury Prevention Research Group, Catholic University San Antonio of Murcia, Campus de los Jerónimos, Guadalupe, 30107 Murcia, Spain

**Keywords:** body composition, body mass index, blood pressure, cardiorespiratory fitness, diet habits, Vo2max

## Abstract

The aim of this study was to evaluate the independent and combined associations between adherence to the Mediterranean diet (AMedDiet), cardiorespiratory fitness (CRF), and different parameters of overweight and obese middle-aged and older adults. Sixty-two participants were enrolled in this cross-sectional study. Fat mass was measured with Dual energy X-ray absorptiometry. AMedDiet and physical activity (PA) were assessed with the PREDIMED and Global PA Questionnaire (GPAQ). Maximal aerobic power was assessed using the 6-min walk test. Systolic (SBP) and diastolic (DBP) blood pressure (BP) were measured with Omron M6, and double product (DP) and mean BP (MBP) were calculated. Kinanthropometry proportionality variables related to obesity were also calculated. Participants with a low CRF as an independent factor or together with a low AMedDiet obtained significantly higher BP, total and trunk fat mass, and proportionality variables (all *p* ˂ 0.0001). According to the multiple nonlinear regression analysis, Vo2max, AMedDiet, and sex explained 53.4% of SBP, with this formula: 238.611 − (3.63*Vo2max) + (0.044*Vo2max^2^) − (13.051*AMedDiet) + (0.68*AMedDiet^2^) + (12.887*sex). SBP and p rediction SBP with the new formula showed a correlation of 0.731 (*p* ˂ 0.0001); showing a difference between the values of −0.278 (*p* = 0.883). In conclusion, CRF as an independent factor and combined with AMedDiet can be associated with BP, body composition, and proportionality in overweight and obese middle-aged and older adults.

## 1. Introduction

Aging is associated with the emergence of chronic diseases, such as cardiovascular disease, overweightness, and obesity [1,2,3]. The adult and older stages are associated with an increase in fat mass [4,5,6], which is linked with a higher incidence of chronic diseases [1,3]. The prevalence of overweightness and obesity in older adults is a worldwide public health problem [7], and the high increase in the number of overweight and obese older adults have resulted in strong concerns about the cost of providing adequate care [8].

Recently, the American Heart Association announced that being older and obese were two of the most dangerous risk factors for uncontrolled hypertension, and defined hypertension as a major risk factor for death and stroke, especially in older years [9]. Also, blood pressure (BP) has been reported to be a risk factor for cardiovascular disease for middle-aged and older adults [10].

Being overweight and obese, coupled with a poor physical condition, are related to aging and are also associated with the risk of death from chronic diseases. Therefore, intervention strategies are needed to encourage changes in body composition and physical condition [11,12]. Public health organizations seek to promote and develop healthy interventions strategies (i.e., daily physical activity, exercise, Mediterranean diet (MedDiet), etc.) among middle-aged and older adults, as they are key aspects for the prevention of obesity [13,14]. Among the healthy strategies for managing high BP in overweight and obese adult we find regular physical activity and healthy eating plan [15,16].

The MedDiet is the traditional diet/lifestyle experienced by people residing near the Mediterranean Sea, including countries from Southern Europe, Northern Africa, and the Middle East [17]. The MedDiet has shown to be a good diet for decreasing hypertension in middle-aged and older adults [18]. In addition, a correlation between the MedDiet and blood pressure has been reported in overweight and obese people [19].

The perception of overweightness has been associated with spontaneous weight management, mainly by dieting and/or exercising [20], but less concern about weight and less perception of overweightness seems to be associated with aging. Ignoring and not addressing excess fat can have a negative impact on the lifestyle and health of older people. It is important for middle-aged and older people to understand the need for a healthy lifestyle, to take care of their diet, and to be physically active. For that, physical activity and healthy dietary habits are essential for greater health-related cardiorespiratory fitness (CRF), and together with body composition have been shown to be directly associated with improved health [21]. Physical activity program interventions are simple, cost-effective strategies for delaying the prevention of chronic disease, hypertension, disability, and improving physical function of middle-aged and older adults. [8]. There is strong evidence that regular exercise or chronically increased physical activity, which increases cardiorespiratory fitness (CRF), attenuates the progressive age-related rise in blood pressure (BP) and prevents hypertension [22]. In addition, the role of the MedDiet in the prevention of chronic diseases has been evidenced by numerous studies [23,24,25,26].

Previous studies [27,28,29,30,31] have shown that increased adherence to the MedDiet (AMedDiet) reduces the risk of chronic diseases such as cardiovascular disease, BP, type II diabetes mellitus, cancer, mortality risk, etc., and increases life expectancy and quality of life. Due to the importance of a balanced and healthy diet, along with an adequate level of physical activity, which increases CRF and energy expenditure to prevent overweightness and obesity and to improve health-related quality of life in aging, studies that address this issue and propose solutions or intervention strategies are necessary.

Therefore, the aims of this study were (a) to analyze the connections between age, AMedDiet, cardiovascular variables, and CRF; and (b) to evaluate the independent and combined association of the MedDiet and CRF on BP parameters, body composition, and proportionality of overweight and obese middle-aged and older adults.

## 2. Materials and Methods

### 2.1. Study Design

This cross-sectional study is part of a randomized controlled trial study of subjects recruited for research on the association between MedDiet, physical activity, physical fitness, and body composition on physical and psychological health in older adults. It is registered at the International Standard Randomized Controlled Trial (ISRCTN); (ISRCTN28506133). The cross-sectional study design followed the Strobe Statement.

### 2.2. Participants

The participants were recruited through advertisements on senior centers and presentations in the community of the Region de Murcia (Spain).

A total of 62 participants were recruited (average age: 67.72 ± 8.02). The inclusion criteria were: (a) Having a body mass index between 25 and 29.9 (overweight) or between 30 and 34.9 (obese), (b) women and men over 50 years of age, and (c) who were physically independent. Exclusion criteria were: (a) Physical limitations or musculoskeletal injuries that could affect testing of performance, and (b) taking hormonal or steroid or non-steroidal anti-inflammatory medications.

Sample size and power were established in connection with a standard deviation for Vo2max from previous data [32]. The sample size for this study consisted of 62 participants, providing a power of 95% and a significance level of α = 0.05; an estimated error of 1.54 mL/kg/min is reported. The Rstudio 3.15.0 software was used to establish the sample size.

### 2.3. Ethics Approval and Consent to Participate

The study obtained approval on CE111908 by the Catholic University of Murcia ethics committee on research and in accordance with the Declaration of Helsinki. All patients signed written informed consent forms prior to participation in the study. This study was conducted at a Sport Science laboratory.

### 2.4. Variables and Instruments

The same researchers performed all the measurements in a single session between 10:00 and 14:00 h. The participants were examined barefoot with the temperature of laboratory standardized at 24 °C. Before the test measurement, the participants did not perform warm-up or stretching exercises. There was a 5-min rest between measurements. In order to establish the reliability of the examiner, prior to the examination, a double-blind study was performed with 30 subjects, obtaining an intraclass correlation coefficient higher than 95%.

Adherence to the Mediterranean Diet (AMedDiet): the PREDIMED test was used, an instrument for dietary evaluation that provides information on the AMedDiet of older adults [33], which was previously validated in the Spanish population [34]. The PREDIMED study is a multicenter, randomized, primary cardiovascular prevention trial conducted in Spain (www.predimed.es). The reliability of this test was verified in a validation test with older Spanish people up to 65 years of age [35].

The Global Physical Activity Questionnaire (GPAQ) is a modified version of the International Physical Activity Questionnaire (IPAQ) [36], and was developed by the World Health Organization (WHO) in response to a greater interest in the role of physical activity (PA) on health [37,38]. This questionnaire classifies participants according to activity level as the main objective of physical activity measurement, and allows for the study of trends and associations with other types of behavior or health outcomes.

The maximal aerobic power (Vo2max) was assessed using the 6-min walk test (6MWT). The 6MWT has been shown to be a valid, reliable, objective, inexpensive, and easy test used to evaluate cardiorespiratory fitness (CRF) [39,40,41,42,43]. It is a simple test to perform and is better related to the person’s daily life activities than other tests [41,42]. It is used to measure an individual’s sub-maximum aerobic capacity while walking for 6 min. It is suggested that this test should be performed on a flat surface that allows walking for 20 to 30 m. The subject should be relaxed and wear comfortable clothing and shoes, and the heart rate (HR) of each subject was recorded with a POLAR 400 heart rate monitor just before the start of the test and just after the end. The route was marked every 5 m and cones were placed at the turning area. The subject walked at a pace appropriate to his/her condition, being able to stop or slow down if he/she was fatigued, resuming as soon as possible. The trainer could motivate the subjects with phrases such as “You are doing well”, and the total meters walked was recorded [44]. Vo2 max was calculated with the following formula for men and women: Vo2 max for men = 110.546 + 0.063 × (6 min walk test) – 0.250 × (age) − 0.486 × (BMI) − 0.420 × (height) − 0.109 x (HR); and Vo2max for women = Vo2max = 22.506 − 0.271 × (weight) + 0.051 × (6 min walk test) − 0.065 × (age) [45]. This test has good reliability (ranging from 0.95 to 0.97) [46].

Blood pressure (BP) was measured in the sitting position on the right arm, and the mean of two recordings at least 3 min apart was recorded. Systolic blood pressure (SBP), diastolic blood pressure (DBP), and pulse rate (PR) were measured using a Omron M6, Vernon Hills, monitor [47]. The double product (DP), consisting of the SBP multiplied by the PR, is an index of myocardial oxygen consumption. DP = Heart rate × systolic blood pressure [48]. Mean blood pressure (MBP) was calculated with the following formula: 1/3 (SBP − DBP) + DBP. MBP is used in clinical practice and allows blood pressure to be used as a single variable. MBP has been shown to be a precursor to cardiovascular risk in adults [10].

Body composition measurements with Dual energy X-ray absorptiometry (DXA) is a method used for body composition measurements for the evaluation of soft tissue composition [49,50]. DXA (QDR 4500A, fan-beam densitometer, Hologic, Waltham, MA, USA) was used for the measurement of trunk and total fat masses and percentages.

Kinanthropometric parameters were measured based on the standards of the International Society for the Advancement on Kinanthropometry (ISAK) [51]. Weight (kg) was evaluated in light clothing without footwear to the nearest 0.1 kg by using an electronic scale, and height (cm) was measured using a stadiometer to the nearest millimeter (Seca 763 digital scale, Birmingham, UK). Biiliocristal and biacromial breaths were measured with a large sliding caliper to the nearest millimeter (Realmet, Barcelona, Spain). Waist girth was measured with an anthropometric measuring tape (W606PM, Lufkin, EE.UU.).

Body mass index (BMI), Ponderal Index (PI), biiliocristal/biacromial ratio, and waist/height ratio (WHR) are parameters related to overweightness and obesity [52,53]. BMI was calculated as weight (kg) divided by height (m) squared [54]. PI was calculated as: weight (g) × 100/(height, cm^3^) [55,56]. Biiliocristal/biacromial ratio was calculated as biiliocristal breath divided by biacromial breath, both in cm [57]. WHR was calculated as waist girth divided by height, both in cm [53].

### 2.5. Statistical Analyses

An analysis of normality of the variables was conducted with the Kolmogorov–Smirnov test and the analysis of sphericity with Mauchly’s test. Data that did not exhibit a normal distribution were subjected to natural log transformation. Data are expressed as means (X) and standard deviations (SD). Pearson’s bivariate correlation coefficients were used to determine the association between age, cardiovascular variables, AMedDiet, physical activity, and cardiorespiratory fitness. For this, the following ranges were established: r < 0.5 for low correlation, 0.5–0.7 for moderate correlation, and >0.7 for high correlation.

Between-group differences (low vs. high AMedDiet; and low vs. high level of CRF) were examined by independent t-tests. Participants were classified based on the AMedDiet (low/high) and CRF (low/high). In order to ensure a balanced distribution between groups (low vs. high AMedDiet and low vs. high CRF), the cut-off points were established based on the mean of each variable (AMedDiet and CRF). The combined effect of CRF and AMedDiet was assessed by the comparison of means (4 groups) based on one-factor analysis of variance (ANOVA), with Bonferroni correction for multiple comparisons.

A X^2^ analysis (categorical variables) was used to analyze the differences between groups. A Cramer’s V post hoc comparison was applied for 2 × 2 tables, and for 2 × n tables a contingency coefficient was applied, showing the value of the statistic and the *p* value. The maximum expected value was 0.707; with an r ˂ 0.3 showing a low association; a moderate association was defined as an r value between 0.3 and 0.5 and a high association was defined as r > 0.5.

Stepwise multiple linear regression models were used to explore the associations between age, sex, CRF, and AMedDiet with cardiovascular variables (DBP, MBP, and DP). The analysis reported three models: Model 1 for CRF, Model 2 for CRF and AMedDiet, and Model 3 for CRF, AMedDiet, and sex. To analyze whether a nonlinear multiple regression models provided the best explanation of the variance, a curvilinear estimation analysis was used to explore the best model association between the dependent and independents variables. A multiple nonlinear regression analysis was performed to analyze the association between the dependent variable with the independent variables.

Statistical significance was set at *p* < 0.05. The statistical analyses were carried out using SPSS (Version 22, SPSS Inc., Chicago, IL, USA).

## 3. Results

Table 1 show the characteristics of the participant.

Table 2 shows the bivariate correlations between age, AMedDiet, physical activity, cardiorespiratory fitness, and correlations between them and BP variables, proportionality, and body composition. Age shows a positive and significant correlation with SBP, biiliocristal/biacromial ratio, and WHR, and a negative correlation with DBP, distance in 6 min test, Vo2max, and PI. AMedDiet was correlated with SBP and MVPA. Vo2max was correlated with age, SBP, MBP, DP, VPA, proportionality, and fat mass.

Table 3 report the result of cardiovascular, proportionality, and body composition according to AMedDiet. Low AMedDiet shows high values SBP, MBP, PR, DP, BMI, and fat mass, although this difference is no significate.

Participants who reported a high CRF showed lower values of SBP, DBP, MBP, DP, BMI, PI, biiliocristal/biacromial ratio, and all the variables related with fat mass, significantly (Table 4).

The analysis between groups of level of CRF + AMedDiet shows that the group with low CRF and low AMedDiet had the highest SBP, MBP, DP, BMI, PI, WHR, and all the variables related to fat mass, significantly. Table 5 shown this and other differences between the variables and groups.

According to the multiple linear regression analysis, Vo2max, AMedDiet, and sex together explained 35.8% of the variation in SBP (Model 3—Table 6). The formula reported for the analysis was: SBP = 2.243 − (0.004*Vo2max) − (0.008* AMedDiet) + (0.047*Sex). The variable prediction SBP was calculated with this formula. The relationship between SBP and prediction SBP was analyzed by means of Student’s t test, showing a correlation of 0.598 (*p* ˂ 0.0001) and a difference between the values of −0.00008 (Table 6).

In order to analyze if a nonlinear multiple regression models provided the best explanation of the variance, a curvilinear estimation shows that the best association between SBP and Vo2max, AMedDiet, and sex was via a quadratic, quadratic, and linear approach, respectively. The predicted formula was: SBP = a − (b*Vo2max) + (c*Vo2max^2^) − (d*AMedDiet) + (e*AMedDiet^2^) + (f*sex). A multiple nonlinear regression analysis was performed as well. Vo2max, AMedDiet, and sex together explained 53.4% of the variation in SBP. The formula found for the analysis was the following: 238.611 − (3.63*Vo2max) + (0.044*Vo2max^2^) − (13.051*AMedDiet) + (0.68*AMedDiet^2^) + (12.887*sex). The relationship between SBP and prediction SBP was analyzed by means of Student’s t test, showing a correlation of 0.731 (*p* ˂ 0.0001) and a difference between the values of −0.278 (*p* = 0.883).

## 4. Discussion

The focus of the present study was to analyze the association of AMedDiet and CRF of various BP components, which can be used to predict cardiovascular diseases in overweight and obese middle-aged and older adults. A strong relationship between DBP, SBP, MBP, and PR, individually or jointly, and cardiovascular disease has been reported in the literature for middle-aged and older adults [10]. Performing regular physical activity and following a healthy eating plan has been recommended for managing high BP and its effects, especially in the overweight and obese [15,16]. The current study found a correlation between CRF and BP components. In fact, middle-aged and older adults with low CRF showed higher SBP, DBP, MBP, and DP than middle-aged and older adults with high CRF. Previous studies have shown that the increase in Vo2max as a result of aerobic exercise training or combined aerobic and resistance training, produces an independent effect on the decrease of resting and exercise BP [58,59]. In fact, the American College of Sports Medicine has recommended primarily aerobic training to reduce BP [60].

Surprisingly, no correlation was found between physical activity and BP components. Stewart et al. [61] also reported in middle-aged and older adults that BP, and especially DBP, had a higher correlation with CRF than with physical activity. This can be attributed to the importance of long-term practice of exercise, with a sufficient training load [62]. However, there was a correlation between CRF and VPA, although not between CRF and MPA, in the current study. In the same way, a correlation between trunk fat and VPA was found, while no correlations with MPA were observed. This is very important due to the importance of trunk fat in BP parameters and cardiovascular disease [58,63]. Previous studies have suggested the superior effect of vigorous exercise for the improvement of CRF, vascular function and body composition in middle-aged and older adults [58,62,64,65]. Other studies have demonstrated that moderate or vigorous physical activity have different associations on key health parameters. It is possible, therefore, that vigorous physical activity has higher positive associations with the health of overweight and obese middle-aged and older adults. Despite these promising results, questions remain, and further research is needed.

Eating habits are a changeable variable with a high association with BP, and specifically the MetDiet has shown considerable benefits against hypertension in middle-aged and older adults [18]. One important finding of the current research was that AMetDiet had a negative correlation with a high SBP and the group with a low AMetDiet showed high BP variables, although no significant differences were found with respect to the high AMetDiet group. A possible explanation for this may be that the importance of the association on BP was the degree of AMedDiet, but not to achieve a categorical classification. In fact, previous studies have also found a correlation between AMedDiet and DBP and SBP [66,67,68] or only SBP in overweight and obese people after adjusting for several potential confounders [19]. This differs from the findings presented here, where the relation between AMetDiet and DBP could be a consequence of the ages of the participants. In accordance with the present results, previous study has demonstrated that SBP increases with age, while DBP decreases [69]. In this line, SBP has been proposed as superior to DBP in the prediction and evaluation of cardiovascular disease in older adults [10,69,70]. Another possible explanation for this is that fat mass can be associated with greater extent DBP than SBP [19]. Future studies on the association of AMetDiet in DBP and SBP dependent on age and fat mass are therefore recommended.

Besides the correlation between and DBP and SBP, a positive correlation was found between age and CRF and proportionality parameters related with obesity. The same findings have been found in previous studies [61,71], which can be consequence of the aging process [4,5,6]. Furthermore, the PR showed a negative correlation with age, in the same line as previous findings [72].

Body composition plays a pivotal and independent role in BP [58,61]. Although body composition is influenced by physical practice and diet habits [58], increased total fat, particularly abdominal fat, are key features of obese and aging [73]. The results of this study determined that overweight and obese middle-aged and older adults showed healthy values in trunk and total fat and in other proportionality parameters related with obesity [74,75] such as BMI, PI, biiliocristal/biacromial ratio, and WHR when having a high CRF. Previous studies have found that aerobic exercise training or combined aerobic and resistance training can change anthropometric parameters and body composition, related to an improvement in BP parameters [58,61]. The same trend was found related with AMedDiet, especially for trunk fat, although differences were not significant. This confirms the results of previous studies whereby body composition changes were higher as related with CRF than with AMedDiet [76,77]. This finding is very interesting, as trunk fat was shown to have an independent association with BP parameters [58], especially with SBP, while BMI or PI, which related to body mass and height, were strongly correlated with DBP change [61].

It is interesting to note that when eating habits and physical condition were combined, overweight and obese middle-aged and older adults with low AMedDiet and low CRF showed higher SBP, MBP, DP, fat mass, BMI, PI, and WHR than the group with a low AMedDiet and high CRF and/or high AMedDiet and high CRF. This confirms that both aspects are associated with BP variables [2,3], body composition, and proportionality variables related to obesity [58,61,74,75], although CRF seems to have somewhat more importance than diet habits [58,61]. A possible explanation for this might be that overweight and obese people, especially during the aging process, have some hormonal and hemodynamic changes which are associated with hypertension [78] and changes in body composition and physiology of fat [79]. Systematic endurance training, which is highly related with CRF [58,59], has an influence on these parameters [80]. In fact, according to the present results, when CRF is low, the AMedDiet is not a relevant factor [76,77]. Despite these promising results, questions remain.

SBP is an important factor of cardiovascular disease in older adults [10,69,70]. In fact, SBP has been proposed as the primary target of antihypertensive therapy [81]. The most interesting finding was that CRF was an aspect with a high association with SBP, followed by the AMedDiet and sex, and together explained 53.4% of the variation in SBP. Previous studies have tried to archive this aim but not in middle-aged and older adults [61,71]. It is not surprising that physical exercise and diet are among the parameters that are most associated with SBP, since these lifestyle factors are highly related to higher BP parameters [15,16]. The other factor was sex, with men showing a higher risk of developing hypertension, which confirms the results of previous studies [82]. A previous study tried to archive a prediction model of SBP for young and adults.

The strengths of this study were its multifactorial focus and the sample characteristics. In fact, it is the first study which associated eating habits, PA, CRF, and obesity parameters with BP components of overweight and obese middle-aged and older adults, and the interaction between these variables. Another strength was that it is the first study that analyzed factors that can explain the variation in SBP of middle-aged and older adults.

Concerning the study limitations, this study did not include some variables that could also be associated with BP such as smoking, drinking alcohol, being stressed, sleep quality and quantity, ingestion of salt, or lipid parameters [71]. In addition, this study is not able to corroborate findings with hormonal data. Furthermore, the physical activity was measured with a questionnaire and not with an objective system such as an accelerometer. Another limitation was that due to its cross-sectional design, causal associations could not be established.

## 5. Conclusions

Middle-aged and older adults who are overweight and obese show the existence of correlations between age, AMedDiet, cardiovascular variables, PA, and CRF. Furthermore, CRF as an independent factor and combined with AMedDiet can influence BP, body composition, and proportionality in middle-aged and older adults who are overweight and obese.

## Figures and Tables

**Table 1:** Characteristics of the participants (*n* = 62).

Variable	X ± SD
Age (year-old)	62.71 ± 8.02
AMedDiet	8.84 ± 2.63
VPA (min)	141.45 ± 360.34
MPA (min)	658.95 ± 647.62
MVPA (min)	800.40 ± 893.04
6 min walk test (m)	536.13 ± 120.36
HR max. in 6 min walk test (bpm)	120.98 ± 17.65
HR mean in 6 min walk test (bpm)	107.24 ± 14.68
Vo2max (mL/kg/min)	27.49 ± 8.40
SBP (mmHg)	130.60 ± 19.81
DBP (mmHg)	79.19 ± 9.80
MBP (mmHg)	96.33 ± 10.34
PR (bpm)	70.81 ± 13.02
DP (bpm*mmHg)	9203.28 ± 1986.76
Height (cm)	158.40 ± 9.95
Weight (kg)	74.65 ± 11.63
BMI (kg/m^2^)	29.68 ± 3.55
PI (gr/cm^3^)	1.88 ± 0.27
Biiliocristal/biacromial ratio	83.64 ± 5.73
WHR	0.90 ± 0.09
Fat mass trunk (kg)	16.55 ± 3.39
Fat mass total (kg)	31.90 ± 6.45
Fat percentage trunk (%)	45.18 ± 4.86
Fat percentage total (%)	43.88 ± 5.38

AMedDiet = adherence to Mediterranean diet; VPA = vigorous physical activity; MPA = moderate physical activity; MVPA = moderate to vigorous physical activity; SBP = systolic blood pressure; DBP = diastolic blood pressure; PR = pulse rate; MBP = mean blood pressure; bpm = beats per minute; HR = heart rate; DP = doubled-product; BMI = body mass index; PI = ponderal index; WHR = waist/height ratio; % = percentage.

**Table 2:** Bivariate correlations between age, cardiovascular variables, adherence to the Mediterranean diet, physical activity, and cardiorespiratory fitness.

Variable	Age	AMedDiet	VPA	MPA	MVPA	6 min Walk Test	Vo2max
Age	-	−0.130	0.058	−0.005	−0.005	−0.480 **	−0.390 **
AMedDiet	−0.139	-	0.223	0.227	0.254 *	0.257	0.245
VPA	−0.117	0.223	-	0.532 **	0.789 **	0.245	0.323 *
MPA	0.058	0.227	0.532 **	-	0.940 **	0.062	0.082
MVPA	−0.005	0.254 *	0.789 **	0.940 **	-	0.143	0.188
6 min walk test	−0.480 **	−0.257	0.245	0.062	0.143	-	0.920 **
Vo2 max	−0.390 **	0.245	0.323 *	0.082	0.188	0.920 **	-
SBP	0.354 **	0.323 *	−0.107	0.005	−0.040	−0.417 **	−0.433 **
DBP	−0.352 **	−0.056	−0.085	−0.106	−0.111	0.009	−0.094
MBP	0.008	−0.258	−0.121	−0.057	−0.091	−0.283 *	−0.353 **
PR	−0.259 *	−0.028	−0.043	−0.018	−0.031	−0.101	−0.127
DP	0.009	−0.256	−0.120	−0.026	−0.068	−0.359 **	−0.400 **
Fat mass/height	0.144	−0.048	−0.211	0.048	−0.051	−0.625 **	−0.801 **
BMI	0.222	−0.111	−0.174	−0.046	−0.104	−0.572 **	−0.700 **
PI	−0.341 **	−0.116	−0.179	−0.004	−0.075	−0.760 **	−0.813 **
Biiliocristal/biacromial ratio	0.349 **	−0.134	−0.070	0.047	0.006	−0.422 **	−0.537 **
WHR	0.477 **	−0.087	−0.122	0.037	−0.023	−0.626 **	−0.623 **
Fat mass trunk	0.029	−0.107	−0.226	−0.090	−0.157	−0.189	−0.417 **
Fat mass total	−0.047	−0.038	−0.177	−0.018	−0.085	−0.256	−0.528 **
Fat% trunk	0.119	−0.132	−0.299 *	0.008	−0.116	−0.509 **	−0.665 **
Fat% total	0.055	−0.028	−0.227	0.080	−0.035	−0.554 **	−0.722 **

* = *p* ˂ 0.05; ** = *p* ˂ 0.01; AMedDiet = adherence to the Mediterranean diet; VPA = vigorous physical activity; MPA = moderate physical activity; MVPA = moderate to vigorous physical activity; SBP = systolic blood pressure; DBP = diastolic blood pressure; MBP = mean blood pressure; PR = pulse rate; DP = doubled-product; BMI = body mass index; PI = ponderal index; WHR = waist/height ratio; % = percentage.

**Table 3:** Cardiovascular, proportionality, and body composition variables according to adherence to the Mediterranean diet (AMedDiet).

Variable	Low AMedDiet (*n* = 31) (X ± DS)	High AMetDiet (*n* = 31) (X ± DS)	*p* Value
SBP (mmHg)	133.61 ± 23.09	127.80 ± 16.07	0.332
DBP (mmHg)	78.75 ± 10.71	79.60 ± 9.04	0.746
MBP (mmHg)	97.04 ± 12.15	95.67 ± 8.48	0.619
PR (bpm)	72.82 ± 10.08	68.93 ± 15.20	0.253
DP (bpm*mmHg)	9666.79 ± 1839.24	8770.67 ± 2051.29	0.085
BMI (kg/m^2^)	30.46 ± 4.00	28.90 ± 2.89	0.084
PI (gr/cm^3^)	1.93 ± 0.32	1.83 ± 0.20	0.137
Biiliocristal/biacromial ratio	84.65 ± 5.08	82.62 ± 6.24	0.166
WHR	0.61 ± 0.08	0.58 ± 0.06	0.152
Fat mass trunk (kg)	17.34 ± 3.21	15.81 ± 3.44	0.085
Fat mass total (kg)	32.83 ± 6.26	31.03 ± 6.61	0.293
Fat percentage trunk (%)	46.08 ± 4.26	44.34 ± 5.29	0.176
Fat percentage total (%)	44.20 ± 5.12	43.59 ± 5.69	0.671

SBP = systolic blood pressure; DBP = diastolic blood pressure; MBP = mean blood pressure; PR = pulse rate; bpm = beats per minute; DP = doubled-product; BMI = body mass index; PI = ponderal index; WHR = waist/height ratio; % = percentage.

**Table 4:** Cardiovascular, proportionality, and body composition variables according to cardiorespiratory fitness (CRF).

Variable	Low CRF (*n* = 31) (X ± DS)	High CRF (*n* = 31) (X ± DS)	*p* Value
SBP (mmHg)	142.22 ± 21.32	123.70 ± 14.90	0.001
DBP (mmHg)	83.04 ± 10.46	77.57 ± 7.66	0.041
MBP (mmHg)	102.77 ± 10.25	92.94 ± 7.54	˂0.0001
PR (bpm)	71.00 ± 12.36	70.20 ± 14.56	0.830
DP (bpm*mmHg)	10050.87 ± 2072.89	8625.70 ± 1801.14	0.012
BMI (kg/m^2^)	32.48 ± 3.42	27.49 ± 1.82	˂0.0001
PI (gr/cm^3^)	2.08 ± 0.26	1.69 ± 0.12	˂0.0001
Biiliocristal/biacromial ratio	85.34 ± 7.22	82.01 ± 4.44	0.042
WHR	0.65 ± 0.06	0.56 ± 0.05	˂0.0001
Fat mass trunk (kg)	18.86 ± 3.12	14.68 ± 2.66	˂0.0001
Fat mass total (kg)	36.26 ± 6.70	28.66 ± 4.55	˂0.0001
Fat percentage trunk (%)	47.25 ± 4.35	43.51 ± 4.97	0.005
Fat percentage total (%)	45.85 ± 5.46	42.40 ± 5.16	0.024

SBP = systolic blood pressure; DBP = diastolic blood pressure; MBP = mean blood pressure; PR = pulse rate; bpm = beats per minute; DP = doubled-product; BMI = body mass index; PI = ponderal index; WHR = waist/height ratio; % = percentage.

**Table 5:** Cardiovascular, proportionality, and body composition variables according to adherence to the Mediterranean diet (AMedDiet) and cardiorespiratory fitness (CRF).

Variable	Low CRF + Low AMedDiet (*n* = 13) (X ± DS)	Low CRF + High AMedDiet (*n* = 11) (X ± DS)	High CRF + Low AMedDiet (*n* = 11) (X ± DS)	High CRF + High AMedDiet (*n* = 19) (X ± DS)
SBP (mmHg)	150.83 ± 20.64 ^a,b^	132.82 ± 18.57	121.64 ± 16.61	124.89 ± 14.16
DBP (mmHg)	84.08 ± 10.07	81.91 ± 11.24	76.36 ± 8.14	78.26 ± 7.50
MBP (mmHg)	106.33 ± 8.35 ^a,b^	98.88 ± 11.07	91.45 ± 9.65	93.81 ± 6.14
PR (bpm)	71.08 ± 10.60	70.91 ± 14.58	74.36 ± 11.58	67.79 ± 15.82
DP (bpm*mmHg)	10669.83 ± 1896.11 ^b^	9375.64 ± 2130.04	8980.27 ± 1469.61	8420.42 ± 1976.22
BMI (kg/m^2^)	33.23 ± 3.80 ^a,b^	31.60 ± 2.83 ^c,d^	27.71 ± 2.24	27.37 ± 1.58
PI (gr/cm^3^)	2.11 ± 0.37 ^a,b^	2.03 ± 0.17 ^d^	1.78 ± 0.22	1.72 ± 0.11
Biiliocristal/biacromial ratio	86.30 ± 6.06	84.19 ± 8.55	82.83 ± 4.30	81.53 ± 4.56
WHR	0.66 ± 0.07 ^a,b^	0.63 ± 0.04 ^d^	0.56 ± 0.05	0.56 ± 0.06
Fat mass trunk (kg)	19.62 ± 3.07 ^a,b^	18.03 ± 3.10^d^	14.97 ± 2.03	14.52 ± 6.30
Fat mass total (kg)	37.02 ± 6.34 ^a,b^	35.44 ± 7.28 ^d^	28.98 ± 4.48	28.48 ± 4.70
Fat percentage trunk (%)	47.11 ± 5.42	47.40 ± 3.06	45.12 ± 3.37	42.57 ± 5.56
Fat percentage total (%)	45.08 ± 6.83	46.70 ± 3.56	43.45 ± 3.32	41.79 ± 5.98

SBP = systolic blood pressure; DBP = diastolic blood pressure; MBP = mean blood pressure; PR = pulse rate; bpm = beats per minute; DP = doubled-product; BMI = body mass index; PI = ponderal index; WHR = waist/height ratio; % = percentage; ^a^ = difference between Low CRF + Low AMedDiet and High CRF + Low AMedDiet; ^b^ = difference between Low CRF + Low AMedDiet and High CRF + High AMedDiet; ^c^ = difference between Low CRF + High AMedDiet and High CRF + Low AMedDiet; ^d^ = difference between Low CRF + High AMedDiet and High CRF + High AMedDiet.

**Table 6:** Stepwise multiple linear regression analysis of the relationship of systolic blood pressure (SBP) with cardiorespiratory fitness (CRF), Mediterranean diet (AMedDiet), and sex.

Analysis	R^2^	*p* Value	Included Independent Variables	Standardized Coefficient (β)	*p* Value
Model 1	0.188	0.001	Vo2max	−0.003	0.001
Model 2	0.298	˂0.0001	Vo2max	−0.003	0.004
			AMetDiet	−0.008	0.008
Model 3	0.358	˂0.0001	Vo2max	−0.004	˂0.0001
			AMetDiet	−0.008	0.013
			Sex	0.047	0.034

AMetDiet: Adherence to Mediterranean diet.
